# Improving the use of research evidence in guideline development: 1. Guidelines for guidelines

**DOI:** 10.1186/1478-4505-4-13

**Published:** 2006-11-21

**Authors:** Holger J Schünemann, Atle Fretheim, Andrew D Oxman

**Affiliations:** 1 INFORMA, S.C. Epidemiologia, Istitituto Regina Elena, Via Elio Chianesi 53, 00144 Rome, Italy; 2 Norwegian Knowledge Centre for the Health Services, P.O. Box 7004, St. Olavs plass, N-0130 Oslo, Norway

## Abstract

**Background:**

The World Health Organization (WHO), like many other organisations around the world, has recognised the need to use more rigorous processes to ensure that health care recommendations are informed by the best available research evidence. This is the first of a series of 16 reviews that have been prepared as background for advice from the WHO Advisory Committee on Health Research to WHO on how to achieve this.

**Objectives:**

We reviewed the literature on guidelines for the development of guidelines.

**Methods:**

We searched PubMed and three databases of methodological studies for existing systematic reviews and relevant methodological research. We did not conduct systematic reviews ourselves. Our conclusions are based on the available evidence, consideration of what WHO and other organisations are doing and logical arguments.

**Key questions and answers:**

We found no experimental research that compared different formats of guidelines for guidelines or studies that compared different components of guidelines for guidelines. However, there are many examples, surveys and other observational studies that compared the impact of different guideline development documents on guideline quality.

**What have other organizations done to develop guidelines for guidelines from which WHO can learn?:**

• Establish a credible, independent committee that evaluates existing methods for developing guidelines or that updates existing ones.

• Obtain feedback and approval from various stakeholders during the development process of guidelines for guidelines.

• Develop a detailed source document (manual) that guideline developers can use as reference material.

**What should be the key components of WHO guidelines for guidelines?:**

• Guidelines for guidelines should include information and instructions about the following components: 1) Priority setting; 2) Group composition and consultations; 3) Declaration and avoidance of conflicts of interest; 4) Group processes; 5) Identification of important outcomes; 6) Explicit definition of the questions and eligibility criteria ; 7) Type of study designs for different questions; 8) Identification of evidence; 9) Synthesis and presentation of evidence; 10) Specification and integration of values; 11) Making judgments about desirable and undesirable effects; 12) Taking account of equity; 13) Grading evidence and recommendations; 14) Taking account of costs; 15) Adaptation, applicability, transferability of guidelines; 16) Structure of reports; 17) Methods of peer review; 18) Planned methods of dissemination & implementation; 19) Evaluation of the guidelines.

**What have other organizations done to implement guidelines for guidelines from which WHO can learn?:**

• Obtain buy-in from regions and country level representatives for guidelines for guidelines before dissemination of a revised version.

• Disseminate the guidelines for guidelines widely and make them available (e.g. on the Internet).

• Develop examples of guidelines that guideline developers can use as models when applying the guidelines for guidelines.

• Ensure training sessions for those responsible for developing guidelines.

• Continue to monitor the methodological literature on guideline development.

## Background

The World Health Organization (WHO), like many other organisations around the world, has recognised the need to use more rigorous processes to ensure that health care recommendations are informed by the best available research evidence. This is the first of a series of 16 reviews that have been prepared as background for advice from the WHO Advisory Committee on Health Research to WHO on how to achieve this.

The term guideline can be defined as "a rule or principle that provides guidance to appropriate behaviour" [1]. The Institutes of Medicine define clinical practice guidelines as "systematically developed statements to assist practitioner and patient decision about appropriate health care for specific clinical circumstances". The term "guidelines" in this document should be seen in the broad sense referring to any guideline or recommendation related to healthcare that is relevant to the mission of the WHO, including public health and health policy recommendations. A plethora of guidelines for clinical practice guidelines exist from various organizations, including national and governmental agencies and medical specialty societies. There are fewer guidelines for developing public health and health policy recommendations. We will use the term "guidelines for guidelines" as "guidelines for the development of guidelines and recommendations".

Guidelines for guidelines are important because of reports indicating that the lack of standardized guideline development leads to widely varying recommendations [2]. In this paper we addressed the following questions:

• What have other organizations done to develop guidelines for guidelines from which WHO can learn?

• What should be the key components of WHO guidelines for guidelines?

• What have other organizations done to implement guidelines for guidelines from which WHO can learn?

### What WHO is doing now

An inter-cluster initiative (Guideline Development Group) led by the Evidence and Information for Policy (EIP) cluster produced the "Guidelines for WHO Guidelines" (GWG) as the recommended approach to development of WHO guidelines [3]. The process for developing the WHO document included drafting of the GWG by one group member before revision and approval by the committee. Following approval by the group, this document was reviewed and approved during a cabinet meeting before distribution as a technical cluster note to all WHO members.

The GWG (version March 10, 2003) included the following general proposals for process (see GWG "WHO documents that guide the development, dissemination and implementation of recommendations by WHO" section 5):

"b) choice of [guideline] topics; c) synthesis of the evidence; d) formulation of recommendations; e) dissemination of guidelines". The GWG makes special reference to the National Health and Medical Research Council (NHMRC) of Australia guidelines for guidelines. To accomplish proper guideline development the GWG recommends partnerships within and outside WHO according to a defined set of rules. Specific functions and composition of guideline groups are also described. The GWG also includes advice for the operationalisation of the process (section 6 GWG): a) Selection of partners; b) Organization of guideline groups; c) Process of developing guidelines; d) Guiding values. The committee also produced a self assessment checklist to ensure a consistent level of quality in the guidelines.

Although comprehensive in the coverage of topics, due to brevity most sections of the GWG could not provide the same level of detailed instructions for guideline groups to follow that other organizations provide. Moreover, it is not entirely clear to what extent WHO guideline developers adhere to the GWG, but it appears that few departments have used the GWG [4]. In part this may be a result of a lack of a more detailed handbook that WHO guidelines development committees could follow, although there are a number of other possible explanations.

### What other organisations are doing?

The use and quality of guidelines for guidelines varies across organizations that develop guidelines. There is not an accepted international standard for guideline development. However, there are several specific and detailed examples of methods adopted by other organizations. Some of these are exemplary because they give detailed guidance and resulted from a thorough process. For example, the National Institute for Health and Clinical Excellence (NICE) and the Scottish Intercollegiate Guideline Network (SIGN), both large government agencies that develop guidelines, have produced comprehensive handbooks that provide guidance for its guideline developers [5,6]. A number of professional organizations have also developed detailed guidance documents that advise their guideline developers about methods including the development of templates [7-10].

We describe the single steps of what other organizations do in regards to guideline development and on what grounds they do it in other articles in this series (see [11] for a list of articles). The steps go from setting priorities for guideline topics to implementation of the guidelines. In addition, literature has emerged from independent groups, such as the Conference on Guideline Standardization [12], that address the critical appraisal of guidelines and suggest the need for guidance for each of these steps of guideline development [13]. For example, one tool (the AGREE instrument) has demonstrated its sensitivity to differentiate higher quality guidelines that followed technical documentation from those of lower quality [14].

## Methods

The methods used to prepare this review are described in the introduction to this series [11]. Briefly, the key questions addressed in this paper were vetted amongst the authors and the ACHR Subcommittee on the Use of Research Evidence (SURE). As a result of prior work in the area of guideline methodology we had knowledge of existing guidelines for guidelines by organisations such as NICE, SIGN, the US Preventive Services Task Force (USPSTF), the New Zealand Guideline Group and the Australian NHRMC as well as professional societies such as the American College of Chest Physicians (ACCP) and the American Thoracic Society (ATS). We attempted to search PubMed, but were unable to devise a search strategy that was both sensitive and reasonably specific.

Given time constraints we avoided duplication with work of others and focused on sources that had systematically compiled relevant literature. We searched databases maintained by the Agency for Healthcare Research and Quality (AHRQ [15]), the Guidelines International Network (GIN [16]), information obtained from prominent organizations and our own files. The AHRQ database (guideline development methodology and guideline structure) is a comprehensive database that included 1205 references to both journal but also non-journal sources. The GIN database included 104 references. While there was overlap we reviewed these citations in detail and evaluated each of these references for relevance. The answers to the questions are our conclusions based on the available evidence, consideration of what WHO and other organisations are doing and logical arguments.

### Findings

We found no experimental research that compared different formats of guidelines for guidelines or studies that compared different components of guidelines for guidelines. However, there are many examples, surveys and other observational studies of the impact of guideline development documents on guideline quality.

### What have other organizations done to develop guidelines for guidelines that WHO can learn from?

Many large organizations that claim to develop evidence based guidelines have produced accessible, transparent and detailed guidelines for guidelines. To make a guideline for guideline credible and acceptable, individuals with expertise in methodology, process and implementation of guidelines were involved in developing a guideline for guideline document. For example, NICE involved not only various internal groups (the national collaborating centres that develop guidelines, NICE patient involvement units, etc.), but also external advisors, including individual academics and governmental institutions (e.g. SIGN) [5]. SIGN and the RAND corporation published a detailed description of the processes involved in producing guidelines for guidelines involving various stakeholders [6,17].

Other guideline developers carefully select the panels that produce guidelines for guidelines ensuring that methodologists and clinicians as well as representatives of the organization are involved. Most specialty societies have included experts and authorities in the relevant fields. While this bears the risk of involving individuals with less methodological and, therefore, relevant training for guideline development, it ensures that individuals who are knowledgeable about the relevant clinical aspects, including ongoing research, are represented and may supports buy-in by users. Aspects focusing on group processes and selection including patient representation are described in other articles in this series [18,19]. Most organizations obtain approval of the final document by a board or other governing body. While no experimental research indicates that providing a source document (e.g. a handbook) for guideline developers improves the quality of guidelines, observational studies suggest that organizations publishing their guidelines for guidelines in the form of reference material produce more methodologically sound guidelines [20].

### What should be the key components of WHO guidelines for guidelines?

We have identified 19 components that are already or should be included in the GWG and that should be described in detail in a handbook or manual for WHO guideline developers. Other reviews in this series will describe these components in greater detail. We list in parenthesis the review that describes the component in more detail and the section of the GWG that has mentioned the component. The sections in the GWG cited below often consist of a single sentence. The components are:

1) Priority setting ([21] and GWG 5b "Choice of topics for development of WHO guidelines")

2) Group composition (and consultations) ([22] and GWG 6A "Organization of guideline groups")

3) Declaration and avoidance of conflicts of interest ([23] and GWG 6B Note 1, annex A)

4) Group processes ([24] and GWG 6C3 "Process of developing guidelines")

5) Identification of important outcomes including cost ([25], not addressed in GWG)

6) Explicit definition of the question and eligibility criteria ([26,27], not addressed in GWG)

7) Type of study designs for different types of questions ([27], not addressed in GWG)

8) Identification of evidence ([27], GWG 6C2 "Undertake a systematic review")

9) Synthesis and presentation of evidence ([28] and GWG 5C "Synthesizing the evidence")

10) Specification and integration of values ([29] and GWG 6D "Guiding values")

11) Making judgments about desirable and undesirable effects ([29] and [30] and GWG 5d "Making recommendations")

12) Taking account of equity ([31], not addressed in GWG)

13) Grading evidence and recommendations ([30] and GWG Annex B)

14) Taking account of costs ([32] and GWG 5d "Making recommendations")

15) Applicability, transferability and adaptation of guidelines ([33] and GWG 5a "A 3 stage process")

16) Structure of reports ([34] and GWG 6C)

17) Methods of peer review ([20] and [32]not addressed in GWG)

18) Planned methods of dissemination & implementation ([35] and GWG 5e "Dissemination of guidelines")

19) Evaluation of the impact of the guideline ([36] and GWG 6C6)

### What have other organizations done to implement guidelines for guidelines from which WHO can learn?

Other prominent guideline developers, such as NICE and SIGN, have ensured that those stakeholders who will become involved in guideline development also take part in the development of the guidelines for guideline. Similarly, obtaining buy-in from regions and country level representatives for GWG before agreeing on and disseminating a revised version is likely to be important. Once WHO reaches agreement on a revised version of the GWG, it should be widely disseminated and made easily available (e.g. on the Internet).

Examples and worksheets should be provided to facilitate implementation of the GWG. In addition, WHO should ensure training sessions for those responsible for developing guidelines. In their survey of 18 prominent international clinical guideline developers, Burgers and colleagues found that almost all guideline programs offer (in some organizations mandatory) training sessions to guideline developers [37]. SIGN, for example, offers a specific software program to guideline panel members and helps them with identifying specific learning needs [6]. SIGN also electronically records the amount of training of individuals who contribute to the guidelines.

The GWG should not be a static document. NICE, for example, has outlined the process for updating its guidelines for guidelines. This specifies that the formal process for updating its manual will begin three years after publication of the original manual. Interim updates may be completed to accommodate small changes outside of the regular renewal process. NICE specifies four criteria that must all be fulfilled to qualify for a minor update: a fundamental stage in the guideline for guideline process is neither added nor removed, a fundamental methods technique or step is neither added nor removed, one or more stakeholders will not obviously be disadvantaged, and the efficiency, clarity or fairness of the process or methodology will be improved. To develop revisions of the GWG, WHO should monitor the methodological literature on guideline development and review updates of other organizations' guidelines for guidelines.

## Discussion

The studies we identified and practical experience suggest that guidelines for guidelines facilitate the development of guidelines. Our review is limited in that it is not a systematic review and is based on our own judgments. However, our review has identified practical advice and components that a handbook or manual that accompany the GWG should include.

Although WHO's leadership has endorsed the GWG, a detailed handbook does not exist. Moreover, implementation of the GWG in WHO guideline programs appears to be very limited [4]. Potential reasons for this shortcoming are discussed elsewhere [4]. Key explanations include a lack of resources, technical (methodological) capacity, knowledge about the GWG, and a tradition of using non-systematic, expert opinion-based approaches [4]; as well as a lack of training and a lack of a more detailed manual.

Other organizations have invested substantial resources into guideline development, including resources to develop and implement guidelines for guidelines. Because WHO has limited resources and because well described processes used by other organizations already exist, WHO can build on existing high quality guidelines for guidelines. As we discuss in another article in this series, WHO should also consider adapting guidelines developed by other organizations, if high quality guidelines already exist [33]. In addition, WHO should consider establishing collaborations with other guideline developers to avoid unnecessary duplication of efforts and use of resources.

### Further work

A systematic review of guidelines for guidelines is unlikely to yield empirical information beyond what we have found in this review, but could provide useful information about what other organisations are doing with respect to key steps in the guideline development process. We do not consider such a review to be a priority for WHO. Similar information has been obtained through surveys [20, 38, 39]. More information about specific questions regarding, for example, processes that are used for updating, implementing and evaluating guidelines for guidelines, is more likely to come from further surveys than from a systematic review.

WHO should develop a handbook or manual that provides detailed information and examples for its guideline developers. This handbook should build on existing work, but will require time and resources. Nonetheless, this is likely necessary to improve the quality of WHO guideline development.

Efforts are needed that ensure guideline developers begin speaking the same "guideline" language and improve the standardisation of the guideline development processes used by WHO. This standardisation would help facilitate the production of guidelines that can easily be adapted to different contexts, and thus reduce global resources spent on guideline development, particularly for low and middle-income countries [33]. WHO should participate in international efforts aimed at improving guidelines for guidelines, and should aim to take a leading role in these efforts in the future.

## Competing interests

ADO and AF work for the Norwegian Knowledge Centre forthe Health Services, an agency funded by the Norwegian government that produces systematic reviews and health technology assessments. All three authors are contributors to the Cochrane Collaboration. ADO and HJS are members of the GRADE Working Group. HJS is documents editor and chair of the documents development and implementation committee for the American Thoracic Society and senior editor of the American College of Chest Physicians' Antithrombotic and Thrombolytic Therapy Guidelines.

## Authors' contributions

HJS prepared the first draft of this article. AF and ADO contributed to drafting and revising it.  All authors have read and approved of the final version of this manuscript.
